# Comprehensive Ophthalmic Evaluation of Clinically Normal Rhesus Monkeys (*Macaca mulatta*): Selected Ophthalmic Diagnostic Tests, Tear‐Film Biochemistry and Serum Correlations, Conjunctival Microbiota and Ophthalmic Artery Haemodynamics

**DOI:** 10.1002/vms3.71277

**Published:** 2026-10-27

**Authors:** Ali Davari, Mohammad Abarkar, Sajad Maleki, Ehsan Mirsadeghi, Mohammad Reza Tabandeh, Iradj Ashrafi Tamai, Mostafa Hajinasrollah, Ahmad Vosough Taghi Dizaj

**Affiliations:** ^1^ Animal Core Facility, Reproductive Biomedicine Research Center Royan Institute for Animal Biotechnology, ACECR Tehran Iran; ^2^ Department of Clinical Science, Faculty of Veterinary Medicine, Karaj Branch Islamic Azad University Karaj Iran; ^3^ Department of Clinical Science, Faculty of Veterinary Medicine, Science and Research Branch Islamic Azad University Tehran Iran; ^4^ Division of Biochemistry and Molecular Biology, Department of Basic Sciences, Faculty of Veterinary Medicine Shahid Chamran University of Ahvaz Ahvaz Iran; ^5^ Stem Cells and Transgenic Technology Research Center Shahid Chamran University of Ahvaz Ahvaz Iran; ^6^ Department of Microbiology and Immunology, Faculty of Veterinary Medicine University of Tehran Tehran Iran; ^7^ Department of Reproductive Imaging, Reproductive Biomedicine Research Center Royan Institute for Reproductive Biomedicine, ACECR Tehran Iran

**Keywords:** colour Doppler imaging, conjunctival microbiota, intraocular pressure, Rhesus monkey, Schirmer tear test, tear film biochemistry, tear film breakup time

## Abstract

**Background:**

The rhesus monkey (*Macaca mulatta*) is an important non‐human primate model in ophthalmic research; however, comprehensive baseline data integrating clinical ophthalmic examinations, tear biochemistry, conjunctival microbiota and ophthalmic artery haemodynamics are limited. This study established normal ophthalmic baseline values and evaluated tear biochemical components and their correlations with serum concentrations in clinically healthy rhesus monkeys.

**Methods:**

Thirty clinically healthy rhesus monkeys (3–11 years; both sexes) underwent comprehensive ophthalmic examination, including blink frequency, palpebral fissure length (PFL), Schirmer tear test‐1 (STT‐1), tear film breakup time (TBUT), intraocular pressure (IOP) in lateral and seated positions, ophthalmic artery colour Doppler ultrasonography and conjunctival bacterial culture. Tear and serum samples were analysed for albumin, glucose, creatinine, urea nitrogen, cholesterol and total protein. The effects of age and sex and correlations between tear and serum biochemical parameters were evaluated.

**Results:**

Mean baseline values were: blink frequency 10.48 ± 2.33 blinks/min, PFL 16.47 ± 1.49 mm, STT‐1 13.93 ± 4.83 mm/min, TBUT 10.50 ± 2.66 s, IOP 22.22 ± 3.90 mmHg (lateral) and 17.34 ± 3.38 mmHg (seated) and ophthalmic artery Doppler indices of PSV 41.65 ± 3.57 cm/s, EDV 19.45 ± 3.64 cm/s, PI 0.76 ± 0.16 and RI 0.53 ± 0.07. No significant differences were observed between eyes or sexes, whereas age significantly affected STT‐1 and PFL. Conjunctival cultures were predominantly Gram‐positive, with *Staphylococcus* spp. and *Bacillus* spp. most frequently isolated. Glucose and total protein differed significantly between tear and serum samples, while tear and serum glucose and urea concentrations were significantly correlated.

**Conclusions:**

These findings establish comprehensive ophthalmic, microbiological, haemodynamic and tear biochemical baseline values for healthy rhesus monkeys, providing a valuable baseline for clinical evaluation, early disease detection and comparative ophthalmic research.

## Graphical abstract

Comprehensive ophthalmic evaluation of clinically healthy rhesus monkeys identified baseline values for selected ophthalmic diagnostic tests, including STT‐1, TBUT, IOP, PFL, blink rate and ophthalmic artery haemodynamics assessed by CDI. Tear‐film biochemical analyses, serum correlations and conjunctival microbiota characterization further expanded the assessment of ocular physiology. These findings provide baseline data to support future clinical and experimental ophthalmic research in rhesus monkeys.

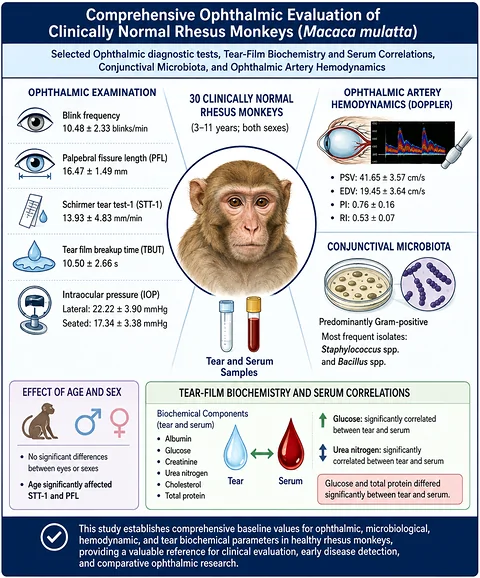

## 1 Introduction

*Macaca mulatta*, commonly referred to as the rhesus monkey, is a species of Old World monkey that belongs to the Cercopithecidae family (Thomasy 2022). Rhesus monkeys share more than 90% of their DNA sequence with humans, making them the preferred non‐human primate (NHP) for ophthalmology research due to their anatomical and physiological similarities to human eyes (Picaud et al. 2019, Fernandes et al. 2023). Rhesus monkeys are typically omnivorous, and their diet includes a wide variety of foods. Depending on their habitat, they consume the most abundant food sources, such as fruits, leaves, eggs, invertebrates like larvae and adult insects, spiders and small vertebrates including fish and crabs (Cooper et al. 2022, Tang et al. 2016). Adult rhesus monkeys usually weigh between 4–10 kg and can live for 25–30 years in captivity, with a maximum recorded lifespan of up to 40 years (Cooper et al. 2022, Colman et al. 2009).

Evaluation of ocular physiology involves various diagnostic tests, together with the assessment of biochemical and microbiological parameters that may reflect underlying health or disease processes. These parameters can provide useful information for researchers and clinicians in the diagnosis and monitoring of ocular diseases; however, available data on these parameters in rhesus monkeys remain limited, highlighting the importance of further investigation.

The Schirmer tear test (STT) is a widely used method for measuring aqueous tear production and has been studied in various wild and exotic species over the years (Oriá, Moncao Silva, et al. 2015, Bezerra et al. 2019). Studies in dogs, horses and humans have demonstrated that eyelid closure can significantly influence STT measurements, highlighting the importance of maintaining a consistent methodology for STT‐1 across measurement sessions (Pe'er et al. 2024, Trbolova and Ghaffari 2017, Kashkouli et al. 2010). Therefore, the present study aimed to evaluate STT‐1 measurements with closed eyelids in rhesus monkeys.

Tear film quality and stability can be assessed using the tear film breakup time (TBUT) test, which is an important indicator of qualitative tear film deficiency and has been evaluated in humans, dogs and cats (Lemp and Hamill 1973, Iwashita et al. 2023, Lim and Cullen 2005). However, limited information exists on TBUT in NHPs, particularly rhesus monkeys, and this knowledge gap needs to be addressed.

Intraocular pressure (IOP) measurement is a crucial aspect of standard ophthalmic examinations and has been studied in various NHPs, including rhesus monkeys (Bezerra et al. 2019, Fernandes et al. 2023, Montiani‐Ferreira et al. 2008, Sigmund et al. 2020, Pletcher et al. 2024, Yu et al. 2009). A review of existing literature indicates that body position affects IOP, a phenomenon that is well documented in humans, cats, dogs and horses (Malihi and Sit 2012, Selk Ghaffari and Arman Gherekhloo 2018, Broadwater et al. 2008, Komáromy et al. 2006). However, this effect has not yet been investigated in rhesus monkeys.

The conjunctival microbiota is an essential component of the eye's defence mechanism, playing a important role in maintaining ocular health by inhibiting the growth of pathogenic microorganisms (Büttner et al. 2019). Understanding the normal conjunctival flora can assist veterinarians in the diagnosis and treatment of ocular diseases. Although the normal conjunctival bacterial flora has been investigated in several NHPs, there is limited information available in scientific literature regarding the normal flora of rhesus monkeys (Bezerra et al. 2019, Montiani‐Ferreira et al. 2008, Oriá et al. 2013).

The tear film is composed mainly of proteins, carbohydrates, lipids and electrolytes, which play essential roles in protecting and maintaining the ocular surface. Any alterations in tear composition can provide valuable insights for diagnosing, predicting and treating eye diseases (Davari et al. 2024, Zong et al. 2022). In the literature, there are only a few studies examining the relationship between clinical chemical variables in serum and tears in companion animals and NHPs (Steinmetz et al. 2019, Raposo et al. 2018, E. Lee et al. 2022, Zapata et al. 2005, Raposo et al. 2020). To date, no research has quantitatively assessed the tear concentrations of albumin, glucose, creatinine, urea nitrogen, cholesterol and total protein in rhesus monkeys and their correlation with serum levels.

Colour Doppler imaging (CDI) is a non‐invasive ultrasound technique commonly used in ophthalmology to assess ocular blood flow dynamics. It provides real‐time visualization of vascular structures and allows measurement of blood flow velocity and resistance indices in retrobulbar blood vessels. Understanding ocular haemodynamics is essential in both clinical and experimental settings, as changes in blood flow are associated with various ophthalmic and systemic diseases (Böhm et al. 2023). Previous studies have investigated CDI in a variety of species, including dog, cat, cow, donkey and human (H. Lee et al. 2002, Vosough 2011, Di Pietro et al. 2015, Wafy et al. 2021, Greenfield and Heggerick 1995); however, limited research has been conducted in NHPs (Netland et al. 1997, Brooks et al. 2007). Therefore, we aimed to evaluate the haemodynamic parameters of the ophthalmic artery (OA) in clinically healthy rhesus monkeys using CDI, thereby establishing baseline data for future comparative and experimental studies.

Although several studies have investigated various aspects of ophthalmology in rhesus monkeys, limited information is available regarding certain diagnostic tests, as well as the proteomic and biochemical profile of their tear film. To address these knowledge gaps, the present study aimed to evaluate various parameters, including blink rate, palpebral fissure length (PFL), STT‐1 with closed eyelids, TBUT, IOP in two body positions and CDI. In addition, the study aimed to characterize the normal conjunctival bacterial flora of the eye and assess the relationship between tear and serum concentrations of selected biochemical analytes across different ages and sexes in anaesthetized clinically healthy rhesus monkeys.

## 2 Methods and Materials

A total of 30 captive‐bred rhesus monkeys (*M. mulatta*), comprising 20 males and 10 females, were included in the study. The animals were 3–11 years old and weighed 3.3–9.2 kg. Of these, 9 animals were classified as juveniles (3–5 years) and 21 as adults (5–11 years). The monkeys used in this study were institution‐owned animals provided by the Royan Primate Research Center (RPRC), Tehran, Iran, and were maintained at the same facility throughout the study period. Each monkey was kept individually in a stainless steel cage, under a 12‐h light/dark cycle, with controlled room temperature and humidity. Their diet consisted of commercial pellets, along with fresh fruits, vegetables and water. From September to November 2024, we conducted a single, thorough ophthalmic examination on 60 eyes from the 30 rhesus monkeys. All experimental procedures involving monkeys were conducted in accordance with the ARVO Statement for the Use of Animals in Ophthalmic and Vision Research and were approved by the Ethics Committee on the Use of Animals at the Royan Institute (Ethics committee registry code: IR.ACECR.AEC.1403.043). Furthermore, all procedures were performed in compliance with the ARRIVE guidelines.

Before the examination and sample collection, the animals were fasted for 12 h. Experienced staff fromRPRC weighed them and then anaesthetized them under the direct supervision of a veterinarian. Anaesthesia was induced by intramuscular injection of 10–15 mg/kg of 10% ketamine (Alfasan, Woerden, the Netherlands) and 0.5 mg/kg of Midazolam 5 mg/mL (Chemidarou, Tehran, Iran). A trained technician and veterinarian continuously monitored the animals throughout the procedure. Each monkey was positioned in dorsal recumbency with its neck extended, allowing its eyes to be oriented horizontally toward the examiner. Following the onset of sedation, blood samples were taken, and a routine physical and basic ophthalmic examination was conducted to screen the animals for any clinical signs of systemic or ocular diseases. We examined both eyes and the surrounding periocular areas with a binocular magnifying loupe to identify macroscopic changes. Monkeys exhibiting systemic diseases, abnormalities in the cornea or anterior chamber, recent use of any topical ophthalmic products within 14 days prior to the study, and a history of ophthalmic diseases were excluded from the study.

To minimize interference with the ophthalmic test results, the thorough ophthalmic examination was delayed until after all tests were completed. Any cases with observed ocular abnormalities were excluded from the study. Each monkey underwent a complete ophthalmic examination, which included an anterior segment assessment using slit‐lamp biomicroscopy (Keeler PSL Classic Portable Slit Lamp; Keeler Ltd.) and a posterior segment evaluation through direct (Pan Optic Ophthalmoscope, Welch Allyn, USA) and indirect ophthalmoscopy, along with a 2.2 Pan Retinal condensing lens (Volk Pan‐Retinal, Volk Optical, USA). All procedures were performed once in the morning (between 8 AM and 12 PM) in the same examination room with controlled ambient temperature and humidity. Ocular measurements were performed at a consistent time interval after induction to minimize variability. The following diagnostic tests were carried out on both eyes of each monkey in a specific sequence: (a) STT‐1; (b) conjunctival swab for microbiological bacterial analysis; (c) TBUT; (d) IOP measurement; (e) PFL; (f) CDI; and finally, (g) measurement of blink rate frequency during a resting state on a separate day. To ensure consistency in results, all ophthalmic tests were conducted by the same evaluator. After completion of all procedures, animals were carefully monitored during recovery for signs of ocular discomfort or general distress. Monitoring continued until normal behaviour, and feeding patterns resumed. The team promptly addressed any adverse effects according to established veterinary guidelines.

### 2.1 Schirmer Tear Test

A standardized sterile strip (Optitech, Allahabad, India) was placed into the ventrolateral conjunctival fornix of the right eye while the monkeys were in a seated position and the eyelids were closed for 1 min to perform a STT‐1 (Figure 1B). Tear production was measured in mm/minute immediately after the 60‐s interval, using a digital stopwatch. Following this, a STT‐1 was conducted on the left eye.

**FIGURE 1:**
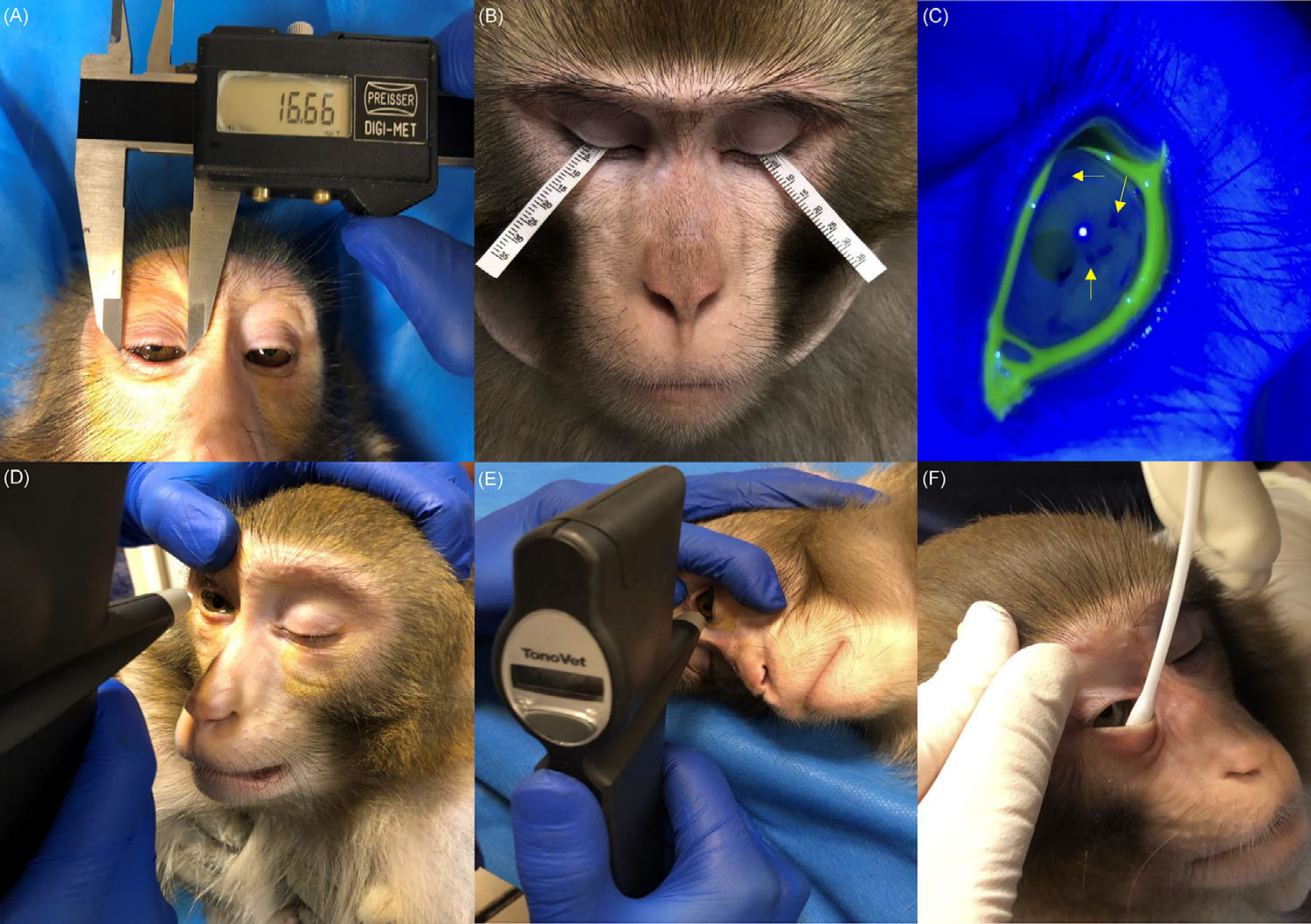
Representative photographs demonstrating selected ocular tests performed in rhesus monkeys (*Macaca mulatta*): palpebral fissure length measurement (A), Schirmer tear test‐1 (B), tear film breakup time (C), intraocular pressure measurement in the seated position (D), intraocular pressure measurement in the lateral position (E) and conjunctival swab collection for microbiological analysis (F).

### 2.2 Microbiological Analysis

Sample collection was conducted using dry sterile swabs (Goodwood medical care Ltd., Jinzhou, China), with separate swabs for each eye and no topical anaesthetics applied to avoid possible interfering with organism growth. The swabs were gently rolled on the surface of the lower conjunctival sac and immediately placed into sterile tubes for transport (Figure 1F). Care was taken to prevent the tip of the swab from contacting the cornea, eyelids, eyelashes or periocular skin; if any of these were touched, the swab was discarded and a new one was used for sampling. The samples were sent to the microbiology laboratory (College of Veterinary Medicine, University of Tehran), in a thermal box with ice packs for microbial analysis. Each swab was inoculated onto blood agar, MacConkey agar and Sabouraud dextrose agar plates. The blood agar and MacConkey agar plates were incubated at 37°C for 48 h. After 48 h of incubation at 37°C, cultures were checked and subcultured if needed. Samples that showed no bacterial growth after 48 h were considered negative. The isolated organisms were identified through/using morphological assessment, Gram staining and biochemical tests in accordance with standard veterinary microbiological methods (Quinn et al. 2011). The Sabouraud dextrose agar plates were incubated at 30°C and examined daily up to 7 days. Fungal identification was performed based on both macroscopic and microscopic characteristics. Samples were considered positive for fungal growth if any colony consistent with fungal morphology appeared on Sabouraud dextrose agar and was confirmed by microscopic examination.

### 2.3 Tear Film Breakup Time

TBUT was evaluated using fluorescein sodium strips (Optitech, Allahabad, India) mixed with 0.3 mL of sterile saline in a 1‐mL syringe (Faghihi and Rajaei 2023). A drop of the fluorescein solution from the syringe was then applied on the dorsal bulbar conjunctiva, after that the eyelids were closed. Once the eyelids were opened, the dorsolateral corneal surface was examined through a cobalt blue filter on a slit‐lamp biomicroscope at 16× magnification. The time from when the eyelids opened until the first dark spot appeared on the green fluorescent tear film, indicating breakup, was recorded using a stopwatch to measure TBUT (Figure 1C). All measurements were conducted in a consistent examination room with controlled ambient temperature and humidity, and no direct airflow. After completing TBUT, the fluorescein was washed out of each eye with eyewash. For data analysis, the average of three measurements per eye was calculated.

### 2.4 Intraocular Pressure

To analyse IOP in rhesus monkeys, we utilized a rebound tonometer (TonoVet; Icare Finland) set to the ‘d’ setting. We only considered measurements that showed no standard deviation (SD), if any measurements were deemed unacceptable or had high SDs, they were repeated. IOP was measured in two different body positions. Approximately 10 min after induction of anaesthesia, the monkeys were placed in right lateral recumbency with minimal restraint, and IOP was measured (Figure 1E). Immediately thereafter, the monkeys were repositioned into a seated posture with gentle restraint of the forelimbs, and IOP was measured again. During the seated measurement, the head was aligned so that the corneal surface was perpendicular to the ground. For statistical analysis, three successful measurements were recorded for each eye. Care was taken to prevent any iatrogenic increases in IOP by ensuring the monkeys' heads remained upright and facing forward, while avoiding any pressure on their heads and necks during the tonometry.

### 2.5 Palpebral Fissure Length

The PFL was assessed in all 30 rhesus monkeys using a stainless steel digital calliper featuring an LCD display, which has an accuracy of ± 0.02 mm (Stoelting, IL, USA) (Figure 1A).

### 2.6 Colour Doppler Imaging

Colour Doppler imaging of rhesus monkeys was performed transpalpebrally using an ultrasound system (M7Vet, Mindray Bio‐Medical Electronics Co. Ltd., Shenzhen, Guangdong, China) equipped with a linear array transducer providing a frequency range of 4–12 MHz. To evaluate the OA, each monkey was placed in dorsal recumbency, and a volume of ultrasound gel applied to the closed eyelids. The transducer was positioned horizontally across the eyeball, ensuring minimal pressure to avoid vascular compression. The OA was identified near the optic nerve entrance, approximately 10–15 mm posterior to the globe. The sample volume was set to 1.0 mm and placed at the center of the OA flow stream. The insonation angle was maintained below 35°. Pulse repetition frequency (PRF) was adjusted between 3–6 kHz, and gain was optimized to eliminate background noise without signal loss (Figure 2). The ultrasound system automatically calculated peak systolic velocity (PSV), end diastolic velocity (EDV), pulsatility index (PI) and resistive index (RI). The RI can also derived by the equation: (PSV − EDV) / PSV (Xu et al. 2015). The same examiner measured each eye three times, and the mean of the three measurements was used for statistical analysis. At the conclusion of CDI, excess coupling gel was removed with a piece of cotton, and the eyes were rinsed with sterile saline solution.

**FIGURE 2:**
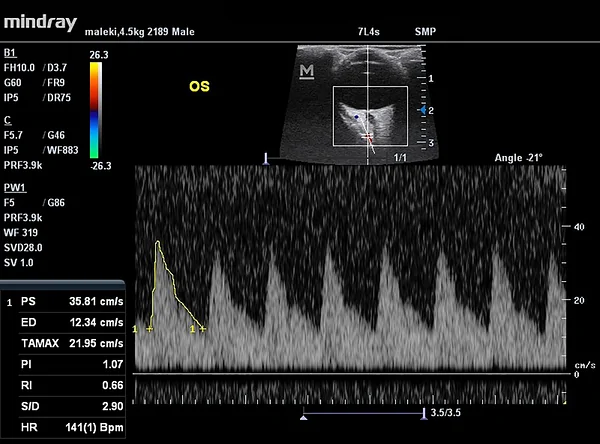
Retrobulbar color Doppler imaging and spectral waveform demonstrating blood flow velocities, including PSV, EDV, PI, RI in the ophthalmic artery of a rhesus monkey. PSV, Peak Systolic Velocity; EDV, End Diastolic Velocity; TAMAX, Time‐Averaged Maximum Velocity; PI, Pulsatility Index; RI, Resistive Index; S/D, Systolic/Diastolic Ratio; HR, Heart Rate.

### 2.7 Blink Rate

Blink rate was measured while the monkeys were in a resting state, characterized by the absence of head movement and a quiet environment. Measurements were performed on a separate day from ocular assessments to avoid potential interference from other procedures. Two trained RPRC staff members independently observed each monkey's eyes from a distance and counted the number of blinks over a 1‐min period. All observations were conducted under consistent ambient conditions.

### 2.8 Tear Film and Serum Collection and Biochemical Analysis

Tear samples from each monkey were collected based on previous studies using Schirmer tear strips (Anonymous, 2024; Anonymous, 2020). Tear sampling was performed immediately after measurement of the STT‐1. Briefly, Schirmer tear strips were remained in the conjunctival fornix until they reached saturation at 30 mm. After saturation, each wetted Schirmer strip was transferred to a 0.5 mL microtube and stored in a −20°C mini cooler until transported to the Royan Proteomics section for tear extraction. The bottom of the microtube was punctured with an 18‐gauge needle, which was then inserted into a 1.5 mL microtube and secured with Parafilm. The microtubes were centrifuged at 12,000 × *g* for 10 min at 4°C. Tear samples obtained from the right and left eyes of the same monkey were pooled after extraction in equal proportions to obtain one representative tear sample per animal and immediately stored at −80°C until further analysis. To assess and recover proteins that may remain adsorbed within the cellulose matrix of Schirmer tear strips after the initial centrifugation, 20 µL of sterile phosphate‐buffered saline (PBS) was added to each used Schirmer strip and then re‐centrifuged under the same conditions (12,000 × *g*, 10 min, 4°C). These PBS‐eluted samples were used only for electrophoretic protein analysis and were not included in the measurements of biochemical parameters. Therefore, the biochemical concentrations reported in this study were not subject to the PBS dilution introduced during the additional elution step.

For blood serum collection, samples were drawn into test tubes without anticoagulant to allow for clotting. After clot formation, serum samples were obtained by centrifugation at 7000 × *g* for 10 min. Concentrations of tear albumin, creatinine, urea, glucose, cholesterol and total protein were measured using commercially available kits (BiorexFars, Iran) following the manufacturer's recommendations using an automated biochemical analyser (Olympus AU40, Japan). The same assay procedures were applied to the tear samples, and all measurements were performed in duplicate. Because the manufacturer's protocols were primarily established for biological fluid samples such as serum/plasma, the results obtained from tear samples were interpreted as measurements in the tear rather than as serum‐equivalent values. Prior to the main experiment, a pilot study was conducted to assess the applicability of the commercial assay kits to tear samples by evaluating all measured analytes and determining whether their concentrations were within the analytical measurement range and above the stated limit of detection (LOD) of the respective kits. Based on the results of this preliminary assessment, the commercial kits were considered suitable for the quantitative measurement of the selected biochemical parameters in the tear samples used in the present study. It is important to note that the sampling of tear film was closely timed with the collection of venous blood samples to ensure consistency in biochemical analysis.

### 2.9 Tear Electrophoresis

The electrophoretic profile of rhesus monkey tears was evaluated using pooled samples from all individuals. Analyses were performed via standard one‐dimensional SDS–PAGE under denaturing and reducing conditions, following the protocol described by Rebouças et al. (2013). A total of 20 µg of protein from the pooled tear sample—Sample 1 (primary eluate obtained after the first centrifugation) and Sample 2 (PBS‐eluted fraction obtained after the second centrifugation)—was loaded onto the 15% polyacrylamide gel. Molecular weights were assessed by comparing the samples to molecular weight standards (PM1700, ExcelBand, SMOBIO Technology Inc., Taiwan). Protein bands were visualized using Coomassie Brilliant Blue staining.

### 2.10 Statistical Analysis

Statistical analyses were performed using GraphPad Prism version 10.4.1 (GraphPad Software, San Diego, CA, USA). Data distribution was assessed using the Kolmogorov–Smirnov test, and since all variables met the assumption of normality, parametric tests were applied. Comparisons between right and left eyes were performed using paired t‐tests. For comparisons between sexes and age groups, the individual animal was considered the statistical unit, and independent‐samples *t*‐tests were used. Similarly, Pearson's correlation analyses between serum and tear biochemical parameters and between ophthalmic variables and age or body weight were performed using individual animal‐level data. Descriptive statistics were calculated for all measurements, and results are presented as mean ± SD. A two‐tailed *p*‐value < 0.05 was considered statistically significant.

## 3 Results

### 3.1 Conjunctival Microbiota

A total of 60 samples from 60 healthy eyes belonging to 30 rhesus monkeys were cultured; 53 samples (88.3%) had positive cultures, while no growth was found in 7 samples (11.7%). Sixty‐six different isolates of bacteria and fungi were found. Of these, 60.6% (*n* = 40) were Gram‐positive, 36.3% (*n* = 24) were Gram‐negative, and 3% (*n* = 2) were fungi. Nine different bacterial genera and 13 bacterial species were isolated. Nine monkeys (30%) shared one bacterial species in both eyes (OU), while 21 monkeys (70%) showed no shared bacteria between the right (OD) and left (OS) eyes. The most commonly isolated Gram‐positive genera were *Staphylococcus* (23/66; 34.8%) and *Bacillus* (9/66; 13.6%). The predominant Gram‐positive species was *S. epidermidis* (11/66; 16.6%). For Gram‐negative isolates, *Escherichia* (6/66; 9%), *Pseudomonas* (5/66; 7.5%) and *Proteus* (5/66; 7.5%) were the most frequently identified genera, with *E. coli* (6/66; 9%) being the leading species. *Candida albicans* (1/66; 1.5%) and *Fusarium solani* (1/66; 1.5%) were the two distinct fungal species found. The results of the bacterial and fungal cultures, along with their isolation frequencies, are presented in Table 1.

**TABLE 1:** Isolates of bacteria and fungi from 60 healthy eyes of 30 rhesus monkeys.

Bacterial species	OD	OS	OU	No (%)
**Gram‐positive**				
*Staphylococcus aureus*	4	4	8	12.1
*Staphylococcus epidermidis*	5	6	11	16.6
*Staphylococcus chromogenes*	3	1	4	6
*Bacillus licheniformis*	5	2	7	10.6
*Bacillus cereus*	—	2	2	3
*Micrococcus luteus*	2	3	5	7.5
*Streptococcus α‐haemolytic*	1	2	3	4.5
**Gram‐negative**				
*Escherichia coli*	2	4	6	9
*Pseudomonas aeruginosa*	2	3	5	7.5
*Enterobacter cloacae*	2	2	4	6
*Proteus mirabilis*	2	3	5	7.5
*Klebsiella aerogenes*	1	—	1	1.5
*Klebsiella pneumoniae*	2	1	3	4.5
**Fungal species**				
*Candida albicans*	—	1	1	1.5
*Fusarium solani*	1	—	1	1.5
**Total**	32	34	66	100

### 3.2 Schirmer Tear Test

In a study of 30 rhesus monkeys, the STT‐1 with closed eyelids showed mean ± SD values of 14.17 ± 5.04 mm/min in the right eye (OD) and 13.70 ± 4.68 mm/min in the left eye (OS), with no significant difference between them (*p* = 0.19). STT‐1 measurements ranged from 5 to 24 mm/min (Table 2). Males showed slightly higher STT‐1 values compared to females (mean difference = 3.42 mm/min, 95% CI = −0.19 to 7.04, *p* = 0.06), suggesting a possible sex‐related trend that did not reach statistical significance. However, age had a significant impact, with adult monkeys exhibiting significantly higher STT‐1 values compared to juveniles (*p* < 0.0001). In addition, STT‐1 was strongly positively correlated with both age (*r* = 0.82) and body weight (*r* = 0.56). A summary of the mean STT‐1 results categorized by sex and age group is presented in Table 3.

**TABLE 2:** Descriptive statistics for selected ophthalmic tests in the rhesus monkey (*Macaca mulatta*).

	Mean	SD	Min	Max	Median
**Ophthalmic tests**					
Schirmer tear test (mm/min)	13.93	4.83	5	24	14
Tear film breakup time (s)	10.50	2.66	6.40	17.10	9.7
Intraocular pressure seated position (mm/Hg)	17.34	3.38	11.33	24.66	17
Intraocular pressure lateral position (mm/Hg)	22.22	3.90	14.66	31	22.83
Palpebral fissure length (mm)	16.47	1.49	14.11	19.69	16.37
Blink rate (blink/min)	10.48	2.33	5	17	10
**Colour Doppler**					
PSV (cm/s)	41.65	3.57	32.39	50.28	41.52
EDV (cm/s)	19.45	3.64	12.34	28.13	18.72
TAMAX (cm/s)	29.68	3.72	21.95	38.78	29.10
PI	0.76	0.16	0.46	1.14	0.75
RI	0.53	0.07	0.39	0.67	0.53
S/D	2.20	0.35	1.63	3.07	2.16

Abbreviations: EDV, end diastolic velocity; PSV, peak systolic velocity; PI, pulsatility index; RI, resistive index; S/D, systolic/diastolic ratio; TAMAX, time‐averaged maximum velocity.

**TABLE 3:** Comparison of mean ± standard deviation and correlation results for ophthalmic parameters across age (juvenile vs. adult) and sex (male vs. female) groups in 30 rhesus monkeys.

Variable	Group	Mean ± SD	*p* value	Correlation with age	Correlation with weight
Schirmer tear test (mm/min)	Male	15.0 ± 4.4	0.06		
	Female	11.6 ± 4.8		*r* = 0.82, *p* < 0.0001*	*r* = 0.56, *p* < 0.001*
	Juvenile	8.5 ± 2.4	< 0.0001*		
	Adult	16.2 ± 3.4			
Tear film breakup time (s)	Male	10.0 ± 1.9	0.17		
	Female	11.4 ± 3.4		*r* = 0.16, *p* = 0.39	*r* = 0.35, *p* = 0.05
	Juvenile	10.5 ± 2.9	0.91		
	Adult	10.4 ± 2.4			
Intraocular pressure seated position (mm/Hg)	Male	17.2 ± 3.4	0.89		
	Female	17.4 ± 2.9		*r* = 0.01, *p* = 0.92	*r* = 0.0, *p* = 0.99
	Juvenile	18.7 ± 3.1	0.11		
	Adult	16.7 ± 3.1			
Intraocular pressure lateral position (mm/Hg)	Male	22.3 ± 3.6	0.79		
	Female	21.9 ± 4.0		*r* = 0.19, *p* = 0.31	*r* = 0.14, *p* = 0.45
	Juvenile	23.0 ± 3.3	0.41		
	Adult	21.8 ± 3.8			
Palpebral fissure length (mm)	Male	16.6 ± 1.6	0.52		
	Female	16.2 ± 1.3		*r* = 0.79, *p* < 0.0001*	*r* = 0.85, *p* < 0.0001*
	Juvenile	15.5 ± 0.9	0.02*		
	Adult	16.8 ± 1.5			
Blink rate (blink/min)	Male	10.7 ± 1.9	0.44		
	Female	10.0 ± 2.5		*r* = 0.0, *p* = 0.99	*r* = 0.11, *p* = 0.53
	Juvenile	10.2 ± 1.3	0.73		
	Adult	10.5 ± 2.4			
**Colour Doppler**					
PSV (cm/s)	Male	41.0 ± 2.8	0.12		
	Female	42.8 ± 2.9		*r* = 0.11, *p* = 0.54	*r* = 0.05, *p* = 0.77
	Juvenile	43.21 ± 3.0	0.06		
	Adult	41.1 ± 2.6			
EDV (cm/s)	Male	19.1 ± 3.0	0.44		
	Female	20.1 ± 3.6		*r* = 0.08, *p* = 0.66	*r* = 0.05, *p* = 0.76
	Juvenile	20.0 ± 4.0	0.60		
	Adult	19.3 ± 3.0			
TAMAX (cm/s)	Male	29.1 ± 3.2	0.22		
	Female	30.6 ± 2.9		*r* = 0.05, *p* = 0.78	*r* = 0.03, *p* = 0.84
	Juvenile	30.7 ± 3.1	0.30		
	Adult	29.4 ± 3.2			
PI	Male	0.76 ± 0.15	0.85		
	Female	0.75 ± 0.15		*r* = 0.15, *p* = 0.40	*r* = 0.10, *p* = 0.59
	Juvenile	0.76 ± 0.19	0.95		
	Adult	0.76 ± 0.13			
RI	Male	0.53 ± 0.06	0.87		
	Female	0.53 ± 0.07		*r* = 0.17, *p* = 0.35	*r* = 0.13, *p* = 0.47
	Juvenile	0.53 ± 0.08	0.98		
	Adult	0.53 ± 0.06			
S/D	Male	2.2 ± 0.33	0.93		
	Female	2.1 ± 0.37		*r* = 0.16, *p* = 0.37	*r* = 0.12, *p* = 0.52
	Juvenile	2.2 ± 0.41	0.92		
	Adult	2.1 ± 0.31			

*Note*: Juvenile (*n* = 9), adult (*n* = 21), male (*n* = 20) and female (*n* = 10). A *p*‐value < 0.05 was considered statistically significant.

Abbreviations: PSV, peak systolic velocity; EDV, end diastolic velocity; TAMAX, time‐averaged maximum velocity; PI, pulsatility index; RI, resistive index; S/D, systolic/diastolic ratio.

### 3.3 Tear Film Breakup Time

The mean TBUT was 10.36 ± 2.27 s for the right eye (OD) and 9.72 ± 1.91 seconds for the left eye (OS), with values ranging from 6.40 to 17.10 s across all eyes (Table 2). Statistical analysis found no significant differences in TBUT between the right and left eyes (*p* = 0.07), age groups (adults vs. juveniles, *p* = 0.91) or sexes (males vs. females, *p* = 0.17) (Table 4).

### 3.4 Intraocular Pressure

Statistical analysis revealed differences in IOP related to body position in rhesus monkeys. IOP was significantly higher when the monkeys were in a lateral recumbent position compared to when they were seated (*p* < 0.0001). The average IOP readings for monkeys in the lateral and sitting recumbent positions were 22.22 ± 3.90 and 17.34 ± 3.38 mmHg, respectively (Table 2). No significant differences were observed between the right and left eyes in both lateral and seated positions. (*p* = 0.81 and 0.17, respectively), and age or sex did not significantly affect IOP (Table 3).

### 3.5 Palpebral Fissure Length

The mean PFL across all monkeys was 16.47 ± 1.49 mm (Table 2). There were no significant differences between the right and left eyes (*p* = 0.83) or between male and female monkeys (*p* = 0.52). However, adult monkeys exhibited a significantly longer palpebral fissure (16.87 ± 1.52 mm) than juveniles (15.55 ± 0.98 mm) (*p* = 0.02). Furthermore, PFL showed a strong positive correlation with both age (*r* = 0.79) and body weight (*r* = 0.85) (Table 3).

### 3.6 Blink Rate

In all monkeys in the study, the mean blink frequency was 10.48 ± 2.33 blink/min, with values ranging from 5 to 17 blink/min (Table 2). No significant differences were observed between males and females (*p* = 0.44) or between juvenile and adult monkeys (*p* = 0.73) (Table 3).

### 3.7 OA Colour Doppler Imaging

CDI of the OA was successfully performed in both eyes of 30 rhesus monkeys. Descriptive statistics for each haemodynamic parameter are presented in Table 2. No significant differences were observed between the right and left eyes for any of the measured Doppler indices (*p* > 0.05). Similarly, no statistically significant differences were detected in PSV, EDV, PI or RI between male and female monkeys (*p* > 0.05). Comparison between juvenile and adult monkeys also revealed no significant differences in any of the measured Doppler indices (*p* > 0.05) (Table 3).

### 3.8 Tear Electrophoretic Profiles

The SDS‐PAGE analysis of the tear electrophoretic protein profile in rhesus monkeys revealed bands with molecular weights varying from under 10 kDa to over 240 kDa. As shown in Figure 3, distinct protein bands were observed in both samples. A total of nine sharp bands were identified, with certain bands appearing more prominent, particularly those within the 10–25 and 55–100 kDa ranges.

**FIGURE 3:**
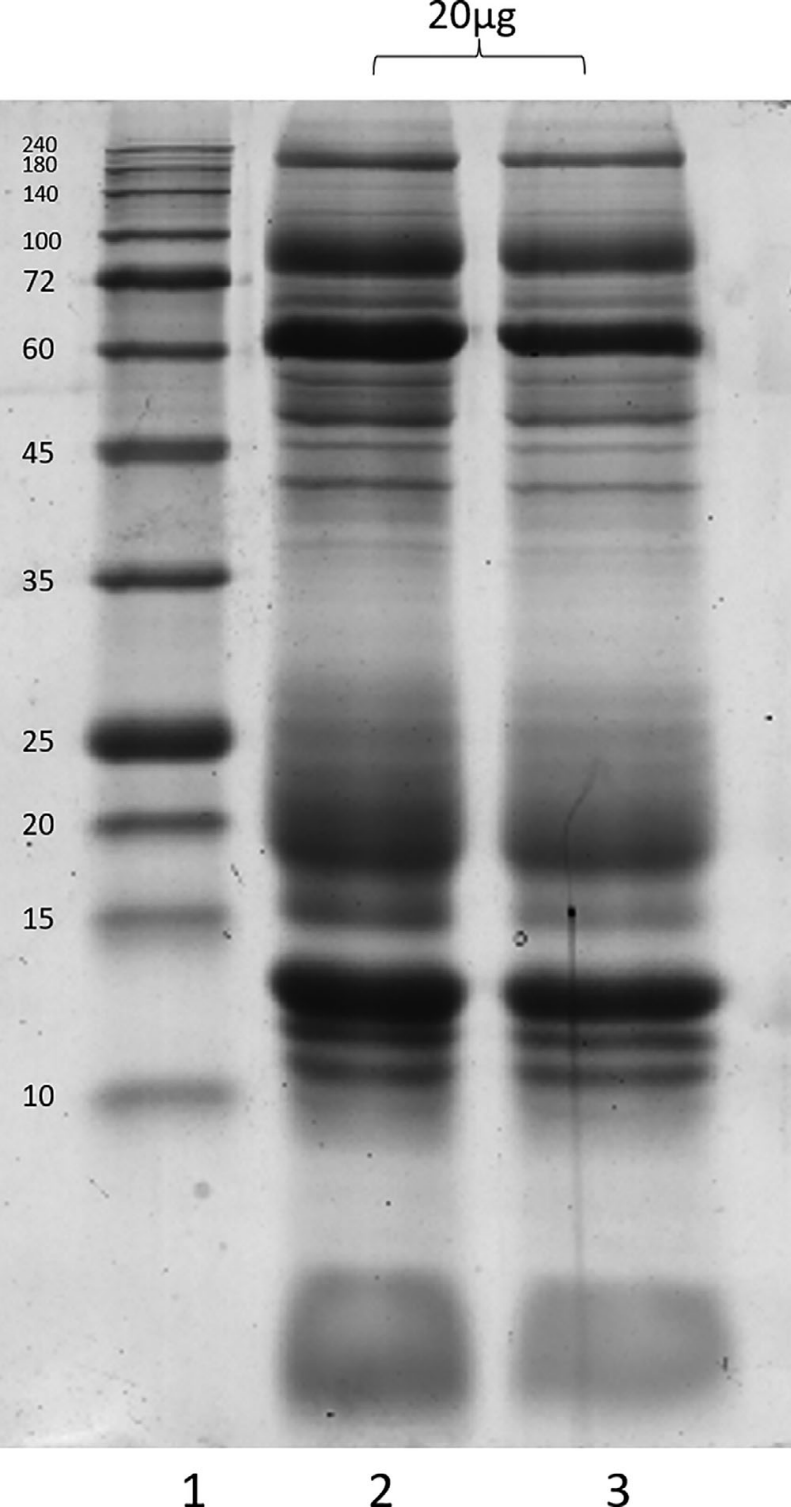
SDS–PAGE profile of pooled tear proteins from rhesus monkeys. Lane 1: prestained molecular weight standards (kDa). Lane 2: 20 µg of total protein from pooled tear samples obtained after the first centrifugation of Schirmer strips (primary eluate; Sample 1). Lane 3: 20 µg of total protein from pooled tear samples obtained after PBS re‐wetting and second centrifugation of the same Schirmer strips (PBS‐eluted fraction; Sample 2). Gels were run under denaturing and reducing conditions on a 15% polyacrylamide gel, and protein bands were visualized by Coomassie Brilliant Blue staining. Full‐length, uncropped gels, are presented in Figure S1.

### 3.9 Comparison and Correlation of Biochemical Parameters in Serum and Tear Samples of Rhesus Monkeys

The mean concentrations of biochemical parameters measured in blood and tear film are summarized in Table 4. Glucose and total protein were the only biochemical parameters that showed variation between groups. Glucose levels varied significantly between age groups in both blood serum (*p* = 0.01) and tear film (*p* = 0.017). Tear film total protein levels also demonstrated significant age‐related variation (*p* = 0.02). No significant differences were observed in any of the other biochemical parameters between males and females or between age groups in either blood serum or tear film samples (*p* > 0.05). A Pearson correlation analysis was conducted to assess the relationship between serum and tear concentrations of the measured parameters. Significant correlations were observed between serum and tear glucose (*r* = 0.89, *p* < 0.0001), and urea (*r* = 0.92, *p* < 0.0001), among others. Detailed correlation plots are shown in Figure 4.

**TABLE 4:** Mean ± SD of biochemical parameters in blood serum and tear film of rhesus monkeys, grouped by sex and age.

Biochemical parameter	Sample type	Overall (*n* = 30)	Male (*n* = 20)	Female (*n* = 10)	Juvenile (*n* = 9)	Adult (*n* = 21)
Glucose (mg/dL)	Blood serum	78.6 ± 20.5	75.7 ± 23.7	84.5 ± 10.6	92.7 ± 9.8	72.5 ± 21.0
	Tear film	9.4 ± 2.4	9.1 ± 2.6	10.0 ± 1.8	11.0 ± 1.5	8.7 ± 2.4
Albumin (g/dL)	Blood serum	4.4 ± 0.3	4.4 ± 0.3	4.5 ± 0.3	4.5 ± 0.2	4.4 ± 0.3
	Tear film	0.2 ± 0.023	0.2 ± 0.021	0.2 ± 0.026	0.2 ± 0.029	0.2 ± 0.020
Cholesterol (mg/dL)	Blood serum	131.8 ± 19.3	134.4 ± 20.4	126.5 ± 16.4	123.6 ± 15.8	135.3 ± 19.9
	Tear film	10.7 ± 2.5	11.0 ± 2.6	10.2 ± 2.4	10.5 ± 2.7	10.8 ± 2.5
Creatinine (mg/dL)	Blood serum	0.93 ± 0.21	0.98 ± 0.22	0.85 ± 0.19	0.84 ± 0.13	0.97 ± 0.23
	Tear film	0.15 ± 0.029	0.15 ± 0.030	0.15 ± 0.029	0.15 ± 0.032	0.15 ± 0.029
Urea (mg/dL)	Blood serum	33.7 ± 7.0	33.0 ± 7.9	35.2 ± 4.7	32.7 ± 6.5	34.1 ± 7.3
	Tear film	38.3 ± 6.6	38.1 ± 7.4	38.8 ± 5.0	37.1 ± 4.9	38.9 ± 7.2
Total protein (g/dL)	Blood serum	6.8 ± 0.3	6.8 ± 0.4	6.9 ± 0.3	6.8 ± 0.2	6.9 ± 0.4
	Tear film	0.7 ± 0.0	0.7 ± 0.0	0.7 ± 0.0	0.6 ± 0.0	0.7 ± 0.0

**FIGURE 4:**
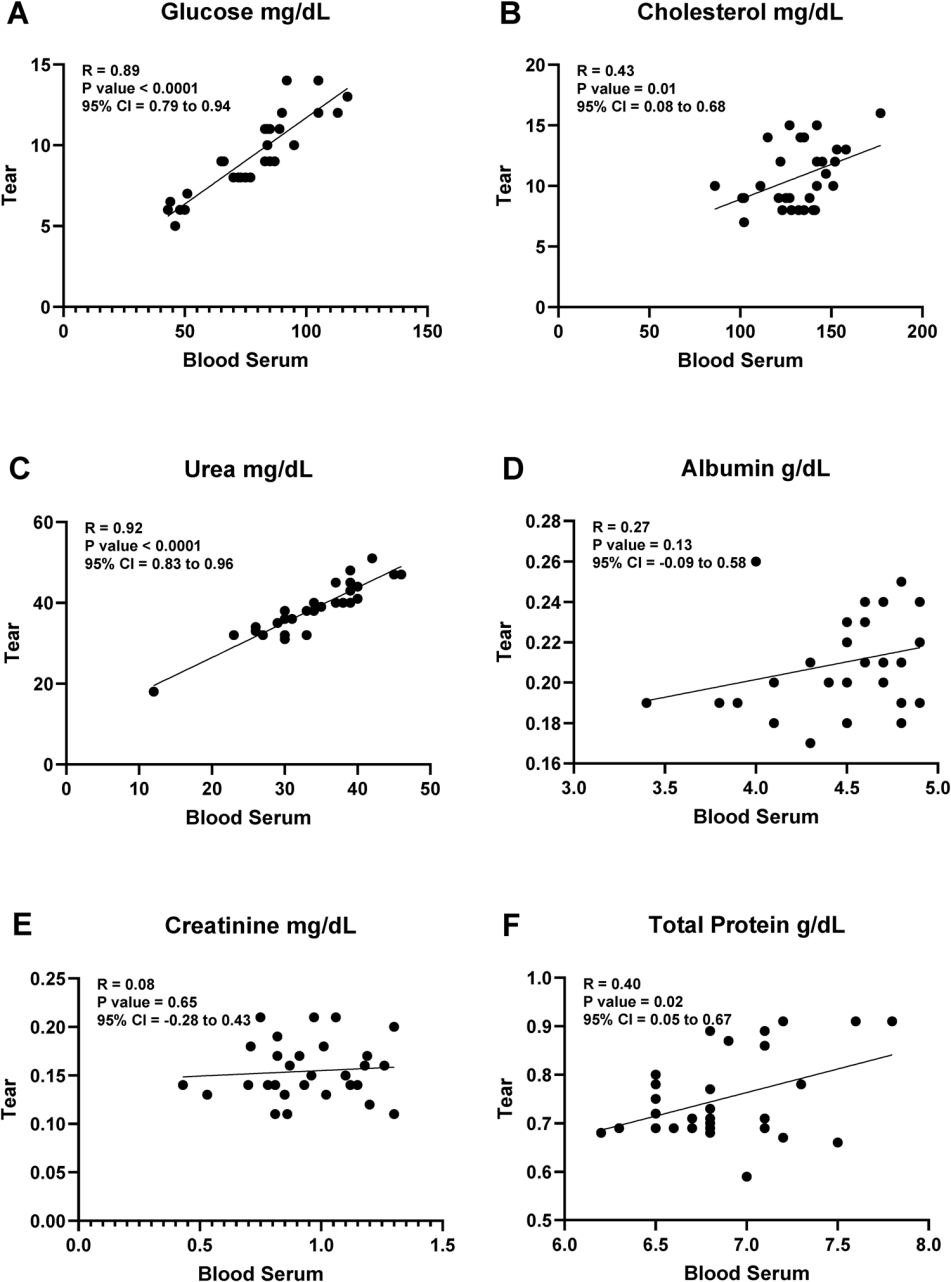
Correlation analysis between serum and tear biochemical factors in rhesus monkeys (n = 30). (A) A very strong positive correlation was observed between serum and tear glucose levels. (B) A moderate positive correlation was found between serum and tear cholesterol levels. (C) Serum and tear urea levels showed a very strong positive correlation. (D) A weak positive correlation was observed between serum and tear albumin levels. (E) No significant correlation was detected between serum and tear creatinine levels. (F) Serum total protein showed a moderate positive correlation with tear levels. Pearson’s correlation test was used, and a p‐value < 0.05 was considered statistically significant.

## 4 Discussion

Establishing baseline biochemical and clinical ocular data is essential for defining reference ranges and improving the diagnosis, monitoring and treatment of ophthalmic diseases. NHPs, particularly the rhesus monkey (*M. mulatta*), are widely regarded as valuable translational models for human ocular disorders because of their anatomical and physiological similarities. Despite their relevance, comprehensive information on normal ocular parameters, tear film composition and related systemic correlations in rhesus monkeys remains limited. The present study addresses this knowledge gap by providing a detailed set of baseline values across a broad range of ophthalmic diagnostic tests, tear film biochemistry, conjunctival microbiota and OA haemodynamics in clinically healthy rhesus monkeys.

A diverse range of bacteria was cultured from most rhesus monkey eyes, with Gram‐positive species predominating. *S. epidermidis* was the most frequent isolate, consistent with reports in other NHPs (marmosets, capuchins) and in humans, dogs, cats and horses, where Gram‐positive flora commonly dominate due to the widespread presence of *Staphylococcus* on skin and mucosa (Bezerra et al. 2019; Oriá et al. 2013). Gram‐negative bacteria, including *Escherichia*, *Pseudomonas* and *Proteus*, were also isolated, in line with findings in howler monkeys and marmosets (Oriá et al. 2013; Galera et al. 2002). *E. coli* was the most common Gram‐negative isolate and is also reported as part of the normal conjunctival flora in capuchins, marmosets, dogs and horses (Bezerra et al. 2019; Oriá et al. 2013; Prado et al. 2005; Wang et al. 2008; Andrew et al. 2003). Their presence likely reflects typical rhesus behaviours—ground contact, faecal and urinary exposure and genital manipulation—along with environmental, seasonal and geographic factors. Two fungal species, *C. albicans* and *F. solani*, were also detected, as previously reported in horses, cattle, cats and dogs (Samuelson et al. 1984). Baseline ocular fungal flora in healthy primates is typically sparse, which may limit detection despite sensitive culture methods. The low prevalence of fungi observed in our study likely reflects transient environmental exposure rather than stable colonization.

The Schirmer tear test (STT‐1) is a key tool in NHP ophthalmic exams, providing an objective assessment of aqueous tear production, which is essential for maintaining corneal and conjunctival health. In this study, mean STT‐1 with closed eyelids was 13.93 ± 4.83 mm/min (range: 5–24), consistent with previously reported values for rhesus and other NHPs, including squirrel and capuchin monkeys (Montiani‐Ferreira et al. 2008; Oriá et al. 2013; Maitchouk et al. 2000). STT‐1 values can be influenced by several factors, including species, anaesthesia, age, environment, diurnal cycles, body weight and stress (Oriá, Gomes Junior, et al. 2015; Bliss et al. 2015). In the present study, the monkeys were anaesthetized with ketamine–midazolam. Previous studies have reported no significant effect of ketamine alone or in combination with other agents on tear production (Jaax et al. 1984; Raposo et al. 2015), whereas isoflurane–midazolam anaesthesia has been associated with reduced tear production (Galera 2021). Therefore, the slightly lower STT‐1 values observed in the present study compared with those reported previously may, in part, be attributable to the anaesthetic protocol, particularly the use of midazolam. Age and body weight were also significant factors influencing STT‐1 values, with juvenile rhesus monkeys exhibiting significantly lower tear production than adults. These findings are consistent with a study in African green monkeys (Pletcher et al. 2024). Furthermore, our results support previous research in dogs, demonstrating a positive correlation between STT‐1 values and both age and weight in rhesus monkeys (de Sampaio et al. 2023).

The mean TBUT in rhesus monkeys was 10.50 ± 2.66 s, closely matching values in healthy humans (10.12 ± 3.90 s) (Gambini et al. 2021). Species‐specific differences have been reported, with cats showing longer TBUT (16.7 ± 4.5 s) and dogs exhibiting wide breed‐dependent variability (5.3 s in Shih Tzus to 24 s in Beagles) (Iwashita et al. 2023; Lim and Cullen 2005). TBUT can be influenced by fluorescein volume, examiner experience and environmental factors such as altitude, humidity, temperature and season. In humans, altitude contributes to variability, while temperature and humidity correlate positively with TBUT (Iwashita et al. 2023; Faghihi and Rajaei 2023). The role of these variables in rhesus monkeys remains unclear. Anaesthesia may also affect TBUT, as significant post‐anaesthetic reductions have been reported in cats (Cullen et al. 2005). Since our measurements were obtained under anaesthesia, TBUT values may not fully represent baseline tear film dynamics. No correlation was found between TBUT and age or body weight in rhesus monkeys, whereas in dogs age correlates negatively with TBUT, and in cats only a weak association has been reported, suggesting species‐specific age effects (Faghihi and Rajaei 2023; Sebbag et al. 2023).

In this study, the mean IOP in seated rhesus monkeys was 17.34 ± 3.38 mmHg, consistent with a previous report (16.9 ± 0.6–18.1 ± 0.6 mmHg) (Yu et al. 2009). For comparison, human IOP averages ∼12 mmHg with rebound tonometry (Nakakura et al. 2019). Comparable IOPs are reported in African green monkeys (16.85 ± 3.85 mmHg), slightly higher values in capuchins (18.4 ± 3.8 mmHg in *S. apella*; 19.2 ± 4.2 mmHg in *S. xanthosternos*), lower values in *S. libidinosus* (13.3 ± 3.32 mmHg), and 14 ± 4.2 mmHg in anaesthetized chimpanzees (Bezerra et al. 2019; Montiani‐Ferreira et al. 2008; Sigmund et al. 2020; Pletcher et al. 2024; Oriá et al. 2013). Ketamine can elevate IOP in some species (e.g., dogs), but has not been reported to significantly alter IOP in cynomolgus or rhesus monkeys or in humans (Bezerra et al. 2019; Sigmund et al. 2020; Pletcher et al. 2024), supporting its use as a sedative in NHP IOP studies. Ketamine–dexmedetomidine also appears not to affect IOP significantly between awake and sedated capuchins (Sigmund et al. 2020). Midazolam lowers IOP in humans, although isoflurane combined with midazolam (0.5 mg/kg) has not been shown to alter IOP in capuchins (Galera 2021; Fragen and Hauch 1981). Thus, potential anaesthetic effects, particularly those associated with midazolam, should be considered when interpreting IOP measurements in NHP studies. Body position significantly influenced IOP: values were higher in the lateral position (22.22 ± 3.90 mmHg) compared to the seated position (17.34 ± 3.38 mmHg). This finding parallels human studies showing elevated IOP in horizontal (prone or supine) positions, and similar effects of body position have also been reported in dogs and horses (Broadwater et al. 2008; Komáromy et al. 2006; Hunt et al. 2004). However, IOP measurements in the present study were always obtained first in lateral recumbency and subsequently in the seated position, with the second measurement performed immediately after repositioning. Therefore, the observed difference between the two positions may reflect not only the effect of body position but also the elapsed time after anaesthetic induction and potential changes in anaesthetic depth. Because the measurement sequence was not randomized, the independent effects of body position and time under anaesthesia cannot be completely separated. Other factors that may influence IOP include intra‐subject variability, diurnal rhythm, stress during restraint and tonometer type (Pletcher et al. 2024). While age has been reported to correlate positively with IOP in African green monkeys (Pletcher et al. 2024), our study found no significant association between age or body weight and IOP, consistent with findings in *S. libidinosus* (Bezerra et al. 2019).

The mean PFL in rhesus monkeys was measured at 16.47 ± 1.49 mm, which is substantially greater than that of the Black‐tufted marmoset (*Callithrix penicillata*), reported at 7.83 ± 0.72 mm, and closely aligns with the value observed in the African green monkey, with a mean PFL of 15 mm (Pletcher et al. 2024; Lange et al. 2012). These differences underscore the species‐specific anatomical variations among NHPs. Establishing normative data for PFL in rhesus monkeys is particularly valuable, as it provides a critical biometric reference for both clinical assessments and research applications.

The average blink rate in rhesus monkeys was 10.48 ± 2.33 blink/min, consistent with a previous report of 10.5 blink/min (Tada et al. 2013) and within the human range (8.5–17.6 blink/min), indicating physiological similarity in blink dynamics (Sebbag and Mochel 2020). In contrast, marmosets show markedly lower rates (40.54 blink/10 min; ∼4.05 blink/min) (Lange et al. 2012). Other species include cats (∼5 blink/min) and dogs (14.2 blink/min) (Oksa‐Minaļto et al. 2023). Such interspecies differences likely reflect variations in ocular surface sensitivity, tear film composition and tear film stability (Sebbag and Mochel 2020). Blinking is essential for maintaining ocular surface integrity by evenly distributing the tear film, thereby supporting surface health and vision.

This study established baseline values and described the colour Doppler ultrasonographic features of the OA in healthy rhesus monkeys. CDI enabled reliable, noninvasive evaluation of retrobulbar vessels, clinically relevant for diagnosing and monitoring ischemic or non‐ischemic vascular occlusions, optic neuropathy and orbital lesions (Wafy et al. 2021). Pulsed‐wave Doppler provided quantitative measures: PSV, EDV, RI and PI, commonly used in human and veterinary medicine to assess vascular integrity and perfusion. RI reflects downstream resistance, while PI characterizes pulsatility and distinguishes arterial from venous flow; physiologic RI values range from 0 (*minimal resistance*) to 1 (*marked resistance*) (Di Pietro et al. 2015). No significant differences were found between right and left eyes, sexes or age groups, consistent with studies in dogs, cats and cattle (E. Lee et al. 2002; Vosough 2011; Di Pietro et al. 2015). In contrast, age influences Doppler indices in humans, and both sex and maturity affect PSV and blood flow in donkeys (Wafy et al. 2021; Greenfield and Heggerick 1995). The OA, located in the deep ventral globe, was readily visualized and yielded the most reproducible results, supporting its value as a reference vessel (Netland et al. 1997). Methodologically, anaesthetic protocols may affect systemic pressure and ocular blood flow, as reported in other species (Di Pietro et al. 2015), and should be considered in future studies.

The electrophoretic profile of rhesus monkey tear proteins analysed by one‐dimensional SDS‐PAGE showed a wide molecular weight distribution, with prominent bands at ∼10–20, ∼25–60 and ∼72–240 kDa, as well as minor bands above 240 kDa. The profiles of the primary tear eluate (Sample 1) and PBS‐eluted fraction (Sample 2) displayed highly similar banding patterns, suggesting that the second extraction step recovered proteins that remained associated with the Schirmer strips rather than a distinctly different protein fraction. Compared with capuchins, rhesus monkeys exhibited more prominent and minor bands spanning < 10 kDa to > 240 kDa (Raposo et al. 2018). Rhesus monkey tear protein profiles also showed similarities to those reported in humans, including a prominent band near ∼66 kDa and multiple components in the ∼30–45 kDa range, which were absent or less prominent in sheep, cattle and camels (Shamsi et al. 2011). Similar to chimpanzees and cattle, rhesus monkeys also showed additional low‐molecular‐weight bands (< 14 kDa) that have been reported to be less prominent or absent in humans (Bodelier et al. 1993). Bands within the ∼35–60 kDa range fall within the reported molecular weight ranges of several tear proteins, including lactoferrin, immunoglobulins and prealbumin, whereas the ∼217 kDa band falls within the range reported for high‐molecular‐weight tear proteins such as mucins or immunoglobulins (Raposo et al. 2018). However, the identities of these protein bands cannot be confirmed based on molecular weight alone, and further approaches such as mass spectrometry or Western blotting would be required for definitive protein identification. In humans, proteins at approximately 14 and 18 kDa have been identified as lysozyme and lipocalin, respectively, and are involved in antimicrobial defense and lipid‐layer stabilization (Wei 2012). Because the present SDS‐PAGE analysis was performed using pooled tear samples, the observed profiles do not allow assessment of inter‐animal biological variability or individual differences in tear protein composition. Although the PBS‐eluted fraction showed slightly lower band intensities, its overall similarity to the primary tear eluate suggests that sequential extraction can recover additional proteins retained within the Schirmer strips, which may be useful when tear volume is limited in NHPs.

We evaluated glucose, albumin, cholesterol, creatinine, urea and total protein in the tear film of healthy rhesus monkeys and compared them with serum values. Glucose showed a significant age‐related difference in both serum and tear samples, while tear total protein also varied with age, potentially reflecting differences in lacrimal gland function, ocular metabolism or tear film stability. Albumin, cholesterol, creatinine and urea showed no significant variation between age groups or sexes. Correlation analysis revealed strong associations between serum and tear concentrations of urea and glucose, consistent with findings reported in humans, dogs, cats and horses (Steinmetz et al. 2019; E. Lee et al. 2022; Zapata et al. 2005; Kang et al. 1988; Tan et al. 2024). Creatinine showed no significant correlation, paralleling findings in other species (Steinmetz et al. 2019; Zapata et al. 2005; Tan et al. 2024). In contrast, cholesterol showed a moderate positive correlation, differing from findings in capuchins and potentially reflecting species‐specific differences in lipid metabolism or transport (Raposo et al. 2018). Correlations for albumin and total protein were weak to moderate, suggesting that their tear concentrations may be influenced by both systemic and local ocular factors. Tear sampling is relatively simple, rapid and minimally invasive and therefore may offer a useful approach for investigating systemic biochemical markers, although its clinical utility remains to be established. The strong associations observed between serum and tear glucose and urea suggest that these analytes warrant further investigation as potential non‐invasive indicators of systemic biochemical status in rhesus monkeys. However, the present study was conducted in clinically healthy animals and did not assess diagnostic accuracy or performance. Future studies involving animals with altered glucose or renal function, larger populations, longitudinal sampling and appropriate diagnostic validation are needed to determine the clinical applicability of tear‐based biochemical measurements and whether tear sampling could complement, rather than replace, conventional venipuncture.

While this study provides valuable baseline information on ophthalmic parameters in rhesus monkeys, several limitations should be acknowledged. First, we obtained all measurements under anaesthesia. In NHPs, including rhesus monkeys, chemical restraint is often necessary because manual restraint is rarely tolerated and may pose risks to both animal welfare and personnel safety, particularly because of potential defensive or aggressive behaviour. Therefore, the use of mild sedation was necessary to ensure animal safety, minimize stress and allow precise control of head and eye positioning during ophthalmic examinations. Nevertheless, anaesthesia may influence certain physiological and ocular parameters and should be considered when interpreting the present findings. Second, the study population was not evenly balanced by sex or age group. The unequal distribution of males and females, and the restriction to only two age categories (juvenile and adult), may limit the generalizability of the results across the full lifespan of rhesus monkeys. Future studies with larger, more balanced samples across sexes and including geriatric animals would provide a more comprehensive understanding of age‐ and sex‐related variation in ophthalmic parameters. Third, the small volume of tear samples obtainable during each collection, which restricted the number of biochemical analyses that could be performed. Collecting larger tear volumes carries the risk of causing ocular surface damage and sample contamination, making it necessary to work with limited amounts. Consequently, pooled tear samples were used for SDS‐PAGE and clinical biochemistry to ensure sufficient material for analysis. Furthermore, the dilution required for biochemical assays may have reduced the sensitivity for detecting certain factors. Together, these challenges highlight the technical constraints associated with tear film studies and the need for further methodological refinement in future research.

## 5 Conclusion

This study provides baseline data on multiple ophthalmic parameters in clinically healthy rhesus monkeys, including STT, TBUT, IOP, PFL, blink rate, colour Doppler imaging of the OA and conjunctival bacterial flora. These findings provide a comprehensive baseline for the assessment of ocular health in this species. They may serve as a useful reference for veterinary ophthalmologists evaluating rhesus monkeys with ocular disorders. The characterization of conjunctival microbiota and the relationships between biochemical parameters in serum and tear samples further contributes to our understanding of ocular physiology in this species. These baseline data may also provide a foundation for future clinical and experimental studies investigating ocular diseases and related physiological changes in rhesus monkeys.

## Author Contributions

**Ali Davari**: investigation, writing – original draft, methodology, writing – review and editing, resources, data curation. **Mohammad Abarkar**: writing – original draft, writing – review and editing, project administration, supervision, methodology. **Sajad Maleki**: methodology, writing – review and editing, investigation. **Ehsan Mirsadeghi**: writing – review and editing, methodology, investigation. **Mohammad Reza Tabandeh**: writing – review and editing, supervision, project administration, visualization, investigation. **Iradj Ashrafi Tamai**: writing – review and editing, methodology. **Mostafa Hajinasrollah**: conceptualization, investigation, writing – review and editing, methodology, data curation, supervision, project administration. **Ahmad Vosough Taghi Dizaj**: conceptualization, project administration, validation, writing – review and editing, methodology.

## Funding

The authors have nothing to report.

## Ethics Statement

All experimental procedures involving monkeys were conducted in accordance with the ARVO Statement for the Use of Animals in Ophthalmic and Vision Research and were approved by the Ethics Committee on the Use of Animals at the Royan Institute (Ethics committee registry code: IR.ACECR.AEC.1403.043). Furthermore, all procedures were performed in compliance with the ARRIVE guidelines.

## Conflicts of Interest

The authors declare no conflicts of interest.

## Supporting information




**Supporting file 01**: vms371277‐sup‐0001‐Figure1.docx

## Data Availability

Data supporting the findings of this study are available from the corresponding author on reasonable request.

## References

[vms371277-bib-0001] Andrew, S. E. , A. Nguyen , G. L. Jones , and D. E. Brooks . 2003. “Seasonal Effects on the Aerobic Bacterial and Fungal Conjunctival Flora of Normal Thoroughbred Brood Mares in Florida.” Veterinary Ophthalmology 6, no. 1: 45–50. 10.1046/j.1463-5224.2003.00265.x 12641842

[vms371277-bib-0002] Bezerra, K. P. G. , R. B. de Lucena , D. T. Stipp , et al. 2019. “Determination of Baseline Values for Routine Ophthalmic Tests in Bearded Capuchin (*Sapajus libidinosus*).” Journal of Medical Primatology 48, no. 1: 3–9. 10.1111/jmp.12378 30230553

[vms371277-bib-0003] Bliss, C. D. , S. Aquino , and S. Woodhouse . 2015. “Ocular Findings and Reference Values for Selected Ophthalmic Diagnostic Tests in the Macaroni Penguin (*E udyptes chrysolophus*) and Southern Rockhopper Penguin (*E udyptes chrysocome*).” Veterinary Ophthalmology 18: 86–93. 10.1111/vop.12123 24238103

[vms371277-bib-0004] Bodelier, V. M. W. , N. J. Van Haeringen , and P. S. Y. Klaver . 1993. “Species Differences in Tears; Comparative Investigation in the Chimpanzee (*Pan troglodytes*).” Primates; Journal of Primatology 34, no. 1: 77–84.

[vms371277-bib-0005] Böhm, E. W. , N. F. Grauhan , N. Pfeiffer , and A. Gericke . 2023. “Measurement of Retrobulbar Blood Flow and Vascular Reactivity—Relevance for Ocular and Cardiovascular Diseases.” Diagnostics 13, no. 23: 3514. 10.3390/diagnostics13233514 PMC10706662 38066755

[vms371277-bib-0006] Broadwater, J. J. , J. J. Schorling , I. P. Herring , and F. Elvinger . 2008. “Effect of Body Position on Intraocular Pressure in Dogs Without Glaucoma.” American Journal of Veterinary Research 69, no. 4: 527–530. 10.2460/ajvr.69.4.527 18380585

[vms371277-bib-0007] Brooks, D. E. , A. M. Komaromy , M. E. Kallberg , T. Miyabashi , F. J. Ollivier , and G. N. Lambrou . 2007. “Blood Flow Velocity Response of the Ophthalmic Artery and Anterior Optic Nerve Head Capillaries to Carbogen Gas in the Rhesus Monkey Model of Optic Nerve Head Ischemia.” Veterinary Ophthalmology 10, no. S1: 20–27. 10.1111/j.1463-5224.2007.00509.x 17973831

[vms371277-bib-0008] Büttner, J. N. , M. Schneider , J. Csokai , E. Müller , and J. C. Eule . 2019. “Microbiota of the Conjunctival Sac of 120 Healthy Cats.” Veterinary Ophthalmology 22, no. 3: 328–336. 10.1111/vop.12598 30095211

[vms371277-bib-0009] Colman, R. J. , R. M. Anderson , S. C. Johnson , et al. 2009. “Caloric Restriction Delays Disease Onset and Mortality in Rhesus Monkeys.” Science (80) 325, no. 5937: 201–204. 10.1126/science.1173635 PMC2812811 19590001

[vms371277-bib-0010] Cooper, E. B. , L. J. N. Brent , N. Snyder‐Mackler , et al. 2022. “The Natural History of Model Organisms: The Rhesus Macaque as a Success Story of the Anthropocene.” Elife 11: e78169. 10.7554/eLife.78169 PMC9345599 35801697

[vms371277-bib-0011] Cullen, C. L. , C. Lim , and J. Sykes . 2005. “Tear Film Breakup Times in Young Healthy Cats Before and After Anesthesia.” Veterinary Ophthalmology 8, no. 3: 159–165. 10.1111/j.1463-5224.2005.00347.x 15910368

[vms371277-bib-0012] Davari, A. , M. R. Tabandeh , M. Abarkar , and B. Torkaman . 2024. “Tear Cytokines as Potential Markers of Inflammation in Feline Conjunctivitis.” Veterinary Ophthalmology 28: 683–690. 10.1111/vop.13261 39161040

[vms371277-bib-0013] de Sampaio, M. O. B. , B. A. Moore , N. M. de Seabra , et al. 2023. “The Development of the Normal Schirmer Tear Test Results During the Dog's First Year of Life.” Veterinary Ophthalmology 26, no. 3: 184–190. 10.1111/vop.13087 36943769

[vms371277-bib-0014] Di Pietro, P. S. , F. Macri , A. P. Piccionello , M. Ragusa , M. M. De Majo , and M. Pugliese . 2015. “Doppler Ultrasonographic Estimation of Ocular Resistive and Pulsatility Indices in Normal Unsedated Cows.” Large Animal Review 21: 235–237.

[vms371277-bib-0015] Faghihi, H. , and S. M. Rajaei . 2023. “Tear Film Breakup Time and Schirmer Tear Test in Normal Dogs: Effects of Age, Sex, Reproductive Status, Skull Type, and Nasolacrimal Duct Patency.” Veterinary Ophthalmology 26: 72–80. 10.1111/vop.13021 36039507

[vms371277-bib-0016] Fernandes, A. G. , P. Alexopoulos , A. Burgos‐Rodriguez , et al. 2023. “Age‐Related Differences in Ocular Features of a Naturalistic Free‐Ranging Population of Rhesus Macaques.” Investigative Ophthalmology & Visual Science 64, no. 7: 3. 10.1167/iovs.64.7.3 PMC10241312 37261386

[vms371277-bib-0017] Fernandes, A. G. , P. Alexopoulos , A. Burgos‐Rodriguez , et al. 2023. “Age‐Related Differences in Ocular Features of a Naturalistic Free‐Ranging Population of Rhesus Macaques.” Investigative Ophthalmology & Visual Science 64, no. 7: 3. 10.1167/iovs.64.7.3 PMC10241312 37261386

[vms371277-bib-0018] Fragen, R. J. , and T. Hauch . 1981. “The Effect of Midazolam Maleate and Diazepam on Intraocular Pressure in Adults.” Arzneimittelforschung 31, no. 12a: 2273–2275. 7199334

[vms371277-bib-0019] Galera, P. D. 2021. “Effects of Isoflurane and Midazolam on Tear Production and Intraocular Pressure in Capuchin Mon‐Keys (*Sapajus libidinosus*).” EC Ophthalmology 12: 47–54.

[vms371277-bib-0020] Galera, P. D. , M. O. Ávila , C. R. Ribeiro , and F. V. dos Santos . 2002. “Estudo Da Microbiota Da Conjuntiva Ocular de Macacos‐prego (*Cebus apella*‐LINNAEUS, 1758) e Macacos Bugio (*Alouatta caraya*‐HUMBOLDT, 1812), Provenientes do reservatório de Manso, MT, Brasil.” Arquivos do Instituto Biológico 69, no. 2: 33–36.

[vms371277-bib-0021] Gambini, G. , M. C. Savastano , A. Savastano , et al. 2021. “Ocular Surface Impairment after Coronavirus Disease 2019: A Cohort Study.” Cornea 40, no. 4: 477–483. 10.1097/ICO.0000000000002643 33214412

[vms371277-bib-0022] Greenfield, D. S. , P. A. Heggerick , and T. R. Hedges 3rd . 1995. “Color Doppler Imaging of Normal Orbital Vasculature.” Ophthalmology 102, no. 11: 1598–1605. 10.1016/s0161-6420(95)30822-6 9098249

[vms371277-bib-0023] Hunt, K. , R. Bajekal , I. Calder , R. Meacher , J. Eliahoo , and J. F. Acheson . 2004. “Changes in Intraocular Pressure in Anesthetized Prone Patients.” Journal of Neurosurgical Anesthesiology 16, no. 4: 287–290. 10.1097/00008506-200410000-00005 15557832

[vms371277-bib-0024] Iwashita, H. , L. Sebbag , B. C. Leonard , and A. Saito . 2023. “A Review of Diagnostic Tests for Qualitative and Quantitative Tear Film Deficiency in Dogs.” Veterinary Ophthalmology 26: 5–15. 10.1111/vop.13044 36575128

[vms371277-bib-0025] Jaax, G. P. , R. R. Graham , and H. Rozmiarek . 1984. “The Schirmer Tear Test in Rhesus Monkeys (*Macaca mulatta*).” Laboratory Animal Science 34, no. 3: 293–294. 6748608

[vms371277-bib-0026] Kang, J. , G. Fulop , and A. H. Friedman . 1988. “Tear Urea Nitrogen and Creatinine Levels in Renal Patients.” Acta Ophthalmologica 66, no. 4: 407–412. 10.1111/j.1755-3768.1988.tb04032.x 3195321

[vms371277-bib-0027] Kashkouli, M. B. , F. Pakdel , A. Amani , M. Asefi , G. H. Aghai , and K. G. Falavarjani . 2010. “A Modified Schirmer Test in Dry Eye and Normal Subjects: Open Versus Closed Eye and 1‐Minute Versus 5‐Minute Tests.” Cornea 29, no. 4: 384–387. 10.1097/ICO.0b013e3181ba6ef3 20164751

[vms371277-bib-0028] Komáromy, A. M. , C. D. Garg , G.‐S. Ying , and C. Liu . 2006. “Effect of Head Position on Intraocular Pressure in Horses.” American Journal of Veterinary Research 67, no. 7: 1232–1235. 10.2460/ajvr.67.7.1232 16817748

[vms371277-bib-0029] Lange, R. R. , L. Lima , and F. Montiani‐Ferreira . 2012. “Measurement of Tear Production in Black‐Tufted Marmosets (*Callithrix penicillata*) Using Three Different Methods: Modified Schirmer's I, Phenol Red Thread and Standardized Endodontic Absorbent Paper Points.” Veterinary Ophthalmology 15, no. 6: 376–382. 10.1111/j.1463-5224.2012.00998.x 22348283

[vms371277-bib-0030] Lee, E. , S. Kang , J. Shim , et al. 2022. “Quantification of Tear Glucose Levels and Their Correlation With Blood Glucose Levels in Dogs.” Veterinary Medicine and Science 8, no. 4: 1816–1824. 10.1002/vms3.788 PMC9297779 35305285

[vms371277-bib-0031] Lee, H. , D. Chang , Y. Lee , et al. 2002. “Use of Color Doppler Imaging for Determining the Resistive Index of the Medial Long Posterior Ciliary Artery in Clinically Normal Conscious Dogs.” American Journal of Veterinary Research 63, no. 2: 211–214. 10.2460/ajvr.2002.63.211 11843120

[vms371277-bib-0032] Lemp, M. A. , and J. R. Hamill . 1973. “Factors Affecting Tear Film Breakup in Normal Eyes.” Archives of Ophthalmology 89, no. 2: 103–105. 10.1001/archopht.1973.01000040105007 4683600

[vms371277-bib-0033] Lim, C. C. , and C. L. Cullen . 2005. “Schirmer Tear Test Values and Tear Film Break‐Up Times in Cats With Conjunctivitis.” Veterinary Ophthalmology 8, no. 5: 305–310. 10.1111/j.1463-5224.2005.00403.x 16178840

[vms371277-bib-0034] Maitchouk, D. Y. , R. W. Beuerman , T. Ohta , M. Stern , and R. J. Varnell . 2000. “Tear Production After Unilateral Removal of the Main Lacrimal Gland in Squirrel Monkeys.” Archives of Ophthalmology 118, no. 2: 246–252. 10.1001/archopht.118.2.246 10676790

[vms371277-bib-0035] Malihi, M. , and A. J. Sit . 2012. “Effect of Head and Body Position on Intraocular Pressure.” Ophthalmology 119, no. 5: 987–991. 10.1016/j.ophtha.2011.11.024 22341914

[vms371277-bib-0036] Montiani‐Ferreira, F. , G. Shaw , B. C. Mattos , H. H. A. Russ , and R. G. D. Vilani . 2008. “Reference Values for Selected Ophthalmic Diagnostic Tests of the Capuchin Monkey (*Cebus apella*).” Veterinary Ophthalmology 11, no. 3: 197–201. 10.1111/j.1463-5224.2008.00620.x 18435663

[vms371277-bib-0037] Nakakura, S. , E. Mori , Y. Fujio , et al. 2019. “Comparison of the Intraocular Pressure Measured Using the New Rebound Tonometer Icare ic100 and Icare TA01i or Goldmann Applanation Tonometer.” Journal of Glaucoma 28, no. 2: 172–177. 10.1097/IJG.0000000000001138 30689609

[vms371277-bib-0038] Netland, P. A. , S. W. Siegner , and A. Harris . 1997. “Color Doppler Ultrasound Measurements After Topical and Retrobulbar Epinephrine in Primate Eyes.” Investigative Ophthalmology & Visual Science 38, no. 12: 2655–2661. 9375585

[vms371277-bib-0039] Oksa‐Minaļto, J. , D. J. Maggs , J. Akimova , A. Ilgaža , and L. Sebbag . 2023. “Ocular Surface Physiology and Aqueous Tear Secretion in Cats of Diverse Cephalic Conformations.” Veterinary Ophthalmology 26, no. S1: 109–118. 10.1111/vop.13051 36519689

[vms371277-bib-0040] Oriá, A. P. , D. C. Gomes Junior , A. V. D. Oliveira , et al. 2015. “Selected Ophthalmic Diagnostic Tests, Bony Orbit Anatomy, and Ocular Histology in Sambar Deer (*Rusa unicolor*).” Veterinary Ophthalmology 18: 125–131. 10.1111/vop.12221 25273613

[vms371277-bib-0041] Oriá, A. P. , R. M. Moncao Silva , M. H. Pinna , et al. 2015. “Ophthalmic Diagnostic Tests in Captive Red‐Footed Tortoises (*Chelonoidis carbonaria*) in Salvador, Northeast Brazil.” Veterinary Ophthalmology 18: 46–52. 10.1111/vop.12175 24783966

[vms371277-bib-0042] Oriá, A. P. , M. H. Pinna , D. S. Almeida , et al. 2013. “Conjunctival Flora, Schirmer's Tear Test, Intraocular Pressure, and Conjunctival Cytology in Neotropical Primates From Salvador, Brazil.” Journal of Medical Primatology 42, no. 6: 287–292. 10.1111/jmp.12059 23879249

[vms371277-bib-0043] Pe'er, O. , R. Ofri , and L. Sebbag . 2024. “Schirmer Tear Test‐1 With Open or Closed Eyelids: An Evaluation in Brachycephalic and Nonbrachycephalic Dogs.” Veterinary Ophthalmology 28: 21–27. 10.1111/vop.13222 PMC11773680 38654458

[vms371277-bib-0044] Picaud, S. , D. Dalkara , K. Marazova , O. Goureau , B. Roska , and J. A. Sahel . 2019. “The Primate Model for Understanding and Restoring Vision.” PNAS 116, no. 52: 26280–26287. 10.1073/pnas.1902292116 PMC6936588 31871177

[vms371277-bib-0045] Pletcher, J. S. , J. L. Zimmer , C. Liu , A. Beierschmitt , and A. C. Lewin . 2024. “Ocular Examination Findings and Selected Ophthalmic Diagnostic Tests in African Green Monkeys (*Chlorocebus aethiops sabaeus*).” Veterinary Ophthalmology 27, no. 2: 158–169. 10.1111/vop.13132 37442802

[vms371277-bib-0046] Prado, M. R. , M. F. G. Rocha , É. H. S. Brito , et al. 2005. “Survey of Bacterial Microorganisms in the Conjunctival Sac of Clinically Normal Dogs and Dogs With Ulcerative Keratitis in Fortaleza, Ceará, Brazil.” Veterinary Ophthalmology 8, no. 1: 33–37. 10.1111/j.1463-5224.2005.04061.x 15644098

[vms371277-bib-0047] Quinn, P. J. , B. K. Markey , F. C. Leonard , P. Hartigan , S. Fanning , and E. Fitzpatrick . 2011. Veterinary Microbiology and Microbial Disease. John Wiley & Sons.

[vms371277-bib-0048] Raposo, A. C. S. , R. D. Portela , M. Aldrovani , T. D. Barral , D. Cury , and A. P. Oriá . 2020. “Comparative Analysis of Tear Composition in Humans, Domestic Mammals, Reptiles, and Birds.” Frontiers in Veterinary Science 7: 283. 10.3389/fvets.2020.00283 PMC7256680 32528986

[vms371277-bib-0049] Raposo, A. C. S. , R. D. Portela , A. Masmali , et al. 2018. “Evaluation of Lacrimal Production, Osmolarity, Crystallization, Proteomic Profile, and Biochemistry of Capuchin Monkeys′ Tear Film.” Journal of Medical Primatology 47, no. 6: 371–378. 10.1111/jmp.12368 30039860

[vms371277-bib-0050] Raposo, A. C. S. , R. Ofri , D. P. H. Schaffer , et al. 2015. “Evaluation of Ophthalmic and Hemodynamic Parameters in Capuchin Monkeys (*Sapajus* sp.) Submitted to Dissociative Anesthetic Protocols.” Journal of Medical Primatology 44, no. 6: 381–389. 10.1111/jmp.12200 26457384

[vms371277-bib-0051] Rebouças, M. F. , D. Loureiro , B. L. Bastos , et al. 2013. “Development of an Indirect ELISA to Detect *Corynebacterium pseudotuberculosis* Specific Antibodies in Sheep Employing T1 Strain Culture Supernatant as Antigen.” Pesqui Veterinária Bras 33: 1296–1302.

[vms371277-bib-0052] Samuelson, D. A. , T. L. Andresen , and R. M. Gwin . 1984. “Conjunctival Fungal Flora in Horses, Cattle, Dogs, and Cats.” Journal of the American Veterinary Medical Association 184, no. 10: 1240–1242. 6539761

[vms371277-bib-0053] Sebbag, L. , and J. P. Mochel . 2020. “An Eye on the Dog as the Scientist's Best Friend for Translational Research in Ophthalmology: Focus on the Ocular Surface.” Medicinal Research Reviews 40, no. 6: 2566–2604. 10.1002/med.21716 32735080

[vms371277-bib-0054] Sebbag, L. , A. Silva , Á. P. B. Santos , A. C. S. Raposo , and A. P. Oriá . 2023. “An Eye on the Shih Tzu Dog: Ophthalmic Examination Findings and Ocular Surface Diagnostics.” Veterinary Ophthalmology 26: 59–71. 10.1111/vop.13022 36057776

[vms371277-bib-0055] Selk Ghaffari, M. , and A. Arman Gherekhloo . 2018. “Effect of Body Position on Intraocular Pressure in Clinically Normal Cats.” Journal of Feline Medicine and Surgery 20, no. 8: 749–751. 10.1177/1098612X17730706 PMC11104139 28942687

[vms371277-bib-0056] Shamsi, F. A. , Z. Chen , J. Liang , et al. 2011. “Analysis and Comparison of Proteomic Profiles of Tear Fluid From Human, Cow, Sheep, and Camel Eyes.” Investigative Ophthalmology & Visual Science 52, no. 12: 9156–9165. 10.1167/iovs.11-8301 22025569

[vms371277-bib-0057] Sigmund, A. B. , A. C. Cushing , and D. V. H. Hendrix . 2020. “Ophthalmic Findings in 10 Captive, Anesthetized Chimpanzees (*Pan troglodytes*).” Veterinary Ophthalmology 23, no. 4: 760–763. 10.1111/vop.12766 32374078

[vms371277-bib-0058] Steinmetz, A. , U. Jörn , G. U. Oechtering , and R. M. Heilmann . 2019. “Tear Glucose, Creatinine, and Urea Nitrogen Concentrations in Cats With Normal or Increased Plasma Concentrations.” Veterinary Ophthalmology 22, no. 4: 470–476. 10.1111/vop.12615 30288908

[vms371277-bib-0059] Tada, H. , Y. Omori , K. Hirokawa , H. Ohira , and M. Tomonaga . 2013. “Eye‐Blink Behaviors in 71 Species of Primates.” PLoS ONE 8, no. 5: e66018. 10.1371/journal.pone.0066018 PMC3669291 23741522

[vms371277-bib-0060] Tan, W. , S. Boveland , M. A. McMichael , et al. 2024. “Agreement Between Lacrimal Fluid and Serum for Detecting Urea Nitrogen and Creatinine in Dogs.” Journal of the American Veterinary Medical Association 262, no. 12: 1650–1656. 10.2460/javma.24.06.0391 39059431

[vms371277-bib-0061] Tang, C. , L. Huang , Z. Huang , A. Krzton , C. Lu , and Q. Zhou . 2016. “Forest Seasonality Shapes Diet of Limestone‐Living Rhesus Macaques at Nonggang, China.” Primates; Journal of Primatology 57, no. 1: 83–92. 10.1007/s10329-015-0498-7 26530218

[vms371277-bib-0062] Thomasy, S. M. 2022. “Ophthalmology of Primatomorpha: Lemurs, Tarsiers, Monkeys, Apes, and Relatives.” Wild and Exotic Animal Ophthalmology 2: 483–543.

[vms371277-bib-0063] Trbolova, A. , and M. S. Ghaffari . 2017. “Results of the Schirmer Tear Test Performed With Open and Closed Eyes in Clinically Normal Horses.” Acta Veterinaria Scandinavica 59: 1–3. 10.1186/s13028-017-0303-2 PMC5452394 28569172

[vms371277-bib-0064] Vosough, D. 2011. “Pulse Doppler Analysis in External Ophthalmic and the Long Ciliary Ophthalmic Arteries in the Cat.” Iranian Journal of Veterinary Surgery 6, no. 1–2: 9–16.

[vms371277-bib-0065] Wafy, M. N. , E. A. Hassan , A. M. A. El‐Maaty , and A. M. Abu‐Seida . 2021. “B‐Scan Biometry and Color Doppler Ultrasound Imaging of the Eye in Clinically Normal Donkeys (*Equus asinus*): Effect of Laterality, Maturity and Gender.” Journal of Equine Veterinary Science 101: 103419. 10.1016/j.jevs.2021.103419 33993935

[vms371277-bib-0066] Wang, L. , Q. Pan , L. Zhang , Q. Xue , J. Cui , and C. Qi . 2008. “Investigation of Bacterial Microorganisms in the Conjunctival Sac of Clinically Normal Dogs and Dogs With Ulcerative Keratitis in Beijing, China.” Veterinary Ophthalmology 11, no. 3: 145–149. 10.1111/j.1463-5224.2008.00579.x 18435654

[vms371277-bib-0067] Wei, X. E. 2012. Biochemical Studies of the Tear Film in Humans and Rabbits. UNSW.

[vms371277-bib-0068] Xu, S. , S. Huang , Z. Lin , W. Liu , and Y. Zhong . 2015. “Color Doppler Imaging Analysis of Ocular Blood Flow Velocities in Normal Tension Glaucoma Patients: A Meta‐Analysis.” Journal of Ophthalmology 2015, no. 1: 919610. 10.1155/2015/919610 PMC4641947 26634152

[vms371277-bib-0069] Yu, W. , G. Cao , J. Qiu , et al. 2009. “Evaluation of Monkey Intraocular Pressure by Rebound Tonometer.” Molecular Vision 15: 2196. PMC2773735 19898690

[vms371277-bib-0070] Zapata, G. L. , R. M. Britos , M. E. Pintos , E. Dreizzen , N. B. Lausada , and S. Arauz . 2005. “Tear Urea Nitrogen and Creatinine Levels in Horse and Their Correlation With Serum Values.” Veterinary Ophthalmology 8, no. 3: 207–209. 10.1111/j.1463-5224.2005.00392.x 15910376

[vms371277-bib-0071] Zong, Y. , C. Cheng , K. Li , et al. 2022. “Metabolomic Alterations in the Tear Fluids of Patients With Superior Limbic Keratoconjunctivitis.” Frontiers in Medicine 8: 797630. 10.3389/fmed.2021.797630 PMC8804220 35118093

