# Supplementary material for: Comprehensive Ophthalmic Evaluation of Clinically Normal Rhesus Monkeys (Macaca mulatta): Selected Ophthalmic Diagnostic Tests, Tear‐Film Biochemistry and Serum Correlations, Conjunctival Microbiota and Ophthalmic Artery Haemodynamics
Source: Vet Med Sci. 2026 Oct 27;12(6):e71277. doi: 10.1002/vms3.71277 (PMC13712712; doi:10.1002/vms3.71277)
Supplement: Supplementary file 1 [file VMS3-12-e71277-s001.docx]

**Supplementary Figure 1.** Full-length, uncropped SDS–PAGE gel of pooled tear proteins from rhesus monkeys. Lane 1 and 4: prestained molecular weight standards (kDa). Lane 2: 30 µg of total protein from pooled tear samples obtained after the first centrifugation of Schirmer strips (primary eluate; Sample 1). Lane 3: 30 µg of total protein from pooled tear samples obtained after PBS re-wetting and second centrifugation of the same Schirmer strips (PBS-eluted fraction; Sample 2). Lane 5: 20 µg of total protein from pooled tear samples obtained after the first centrifugation of Schirmer strips (primary eluate; Sample 1). Lane 6: 20 µg of total protein from pooled tear samples obtained after PBS re-wetting and second centrifugation of the same Schirmer strips (PBS-eluted fraction; Sample 2). Gels were run under denaturing and reducing conditions on a 15% polyacrylamide gel, and protein bands were visualized by Coomassie Brilliant Blue staining.
